# Complete Revascularisation in Impella-Supported Infarct-Related Cardiogenic Shock Patients Is Associated With Improved Mortality

**DOI:** 10.3389/fcvm.2021.678748

**Published:** 2021-07-09

**Authors:** Andreas Schäfer, Ralf Westenfeld, Jan-Thorben Sieweke, Andreas Zietzer, Julian Wiora, Giulia Masiero, Carolina Sanchez Martinez, Giuseppe Tarantini, Nikos Werner

**Affiliations:** ^1^Department of Cardiology and Angiology, Cardiac Arrest Center, Hannover Medical School, Hanover, Germany; ^2^Division of Cardiology, Pulmonology and Vascular Medicine, Medical Faculty, Cardiac Arrest Center, Heinrich Heine University, Düsseldorf, Germany; ^3^Department of Cardiology, University Heart Center, Bonn, Germany; ^4^Department of Cardiology, University of Padua, Padua, Italy; ^5^Department of Cardiology, Heart Center Trier, Krankenhaus der Barmherzigen Brüder, Trier, Germany

**Keywords:** microaxial flow-pumps, acute heart failure, myocardial infarction, cardiogenic shock, revascularisation

## Abstract

**Background:** Acute myocardial infarction-related cardiogenic shock (AMI-CS) still has high likelihood of in-hospital mortality. The only trial evidence currently available for the intra-aortic balloon pump showed no benefit of its routine use in AMI-CS. While a potential benefit of complete revascularisation has been suggested in urgent revascularisation, the CULPRIT-SHOCK trial demonstrated no benefit of multivessel compared to culprit-lesion only revascularisation in AMI-CS. However, mechanical circulatory support was only used in a minority of patients.

**Objectives:** We hypothesised that more complete revascularisation facilitated by Impella support is related to lower mortality in AMI-CS patients.

**Methods:** We analysed data from 202 consecutive Impella-treated AMI-CS patients at four European high-volume shock centres (age 66 ± 11 years, 83% male). Forty-seven percentage (*n* = 94) had cardiac arrest before Impella implantation. Revascularisation was categorised as incomplete if residual SYNTAX-score (rS) was >8.

**Results:** Overall 30-day mortality was 47%. Mortality was higher when Impella was implanted post-PCI (Impella-post-PCI: 57%, Impella-pre-PCI: 38%, *p* = 0.0053) and if revascularisation was incomplete (rS ≤ 8: 37%, rS > 8: 56%, *p* = 0.0099). Patients with both pre-PCI Impella implantation and complete revascularisation had significantly lower mortality (33%) than those with incomplete revascularisation and implantation post PCI (72%, *p* < 0.001).

**Conclusions:** Our retrospective analysis suggests that complete revascularisation supported by an Impella microaxial pump implanted prior to PCI is associated with lower mortality than incomplete revascularisation in patients with AMI-CS.

## Introduction

Acute myocardial infarction (AMI) is one of the major contributors to cardiogenic shock (CS) (1). The “Should We Emergently Revascularize Occluded Coronaries for Cardiogenic Shock” (SHOCK) trial demonstrated that urgent invasive assessment and revascularization improves long term survival (2). Based on this trial, current society guidelines recommend urgent revascularisation in AMI-CS (3). However, even two decades later, mortality in AMI-CS remains high with almost every second patient dying (2, 4–7).

Mechanical circulatory support (MCS) raised hope to improve outcome in AMI-CS. Although the intra-aortic balloon pump (IABP) was the most frequently used MCS device, it failed to improve survival compared to standard medical therapy (6, 8) and thus is no longer recommended for routine use (Class IIIA in the ESC Guidelines) (3). Today, a variety of more powerful MCS devices are available, including Impella, TandemHeart and extracorporeal membrane oxygenation (ECMO) (9). However, due to lacking prospective randomised data, current guidelines for use of MCS in AMI-CS are based on expert opinion and generally do not favour one system over another (3, 10).

Previous trials investigating Impella pumps in AMI-CS were not adequately powered regarding clinically meaningful outcome differences. Additionally, high proportions of patients with out-of-hospital cardiac arrest (OHCA) had a strong and negative influence on the reported mortality rates, and lacked standardisation of timing of Impella placement (11, 12). Several observational studies reported a positive association of Impella support prior to PCI on mortality, especially in patients who did not suffer cardiac arrest before device implantation (13–19). Whether approaches aiming for early implementation of Impella support in AMI-CS relate to improved outcome is currently investigated in the adequately powered DanGer-Shock trial (20). Since DanGer-Shock will most probably require some more years before data are reported, deciding about the use of MCS is based on individual experience.

While it is nowadays recommended to achieve complete revascularisation in stable AMI patients (21), the historic belief of complete revascularisation in AMI-CS has been challenged by the results of the CULPRIT-SHOCK trial, demonstrating lower mortality in AMI-CS patients receiving culprit-lesion-only compared to multivessel revescularisation (22). A major limitation to more complete revascularisation in that trial was haemodynamic instability during PCI in the multivessel group. Whether complete revascularisation in AMI-CS would be associated with improved outcome if patients were stabilised more rigorously by more liberal use of MCS devices in AMI-CS, has not been determined in a prospective study yet. Recently, the American National Shock Initiative Investigators reported about AMI-CS patients treated by a common strategy of early revascularisation on Impella support. In 198 patients with multivessel disease presenting with AMI-CS, revascularization of non-culprit lesions was associated with similar survival compared with culprit-only PCI (23).

Completeness of revascularisation has been addressed in PCI trials in the past using the residual Syntax score (rS) with a value of 8 or less indicating complete revascularisation (21). While the Syntax score was originally derived from a randomised study excluding AMI patients, it has been widely used in AMI patients and rS demonstrated its prognostic relevance in this particular setting as well (24–28). The more recent publications even used a similar threshold for incomplete revascularisation of rS of 8 with persistent prognostic relevance as (26–28). Most recently, the ACTION Core group from Paris used the rS for their analysis on complete revascularisation in the CULPRIT-SHOCK trial. In their analysis, rS was independently associated with early and late mortality (29).

In order to provide more detailed insight into that matter, we collected observational data from four shock centres running Impella programs and report about a total of 202 AMI-CS patients treated with Impella microaxial flow-pumps in clinical routine. We compared 30-day mortality in those patients in relation to completeness of revascularisation using residual syntax score as a previously investigated surrogate for completeness of revascularisation (21).

## Methods

### Study Design

This was a retrospective, observational analysis that included data from all patients undergoing implantation of an Impella CP microaxial flow-pump in AMI-CS between 2012 and 2018 in all four centres when complete revascularisation during the index procedure was the intended strategy based on previous guideline recommendations (30). De-identified data were entered into a combined database. All data were collected in accordance with the Declaration of Helsinki and approved by the local ethics committee of each centre.

In general, all participating centres use algorithms for AMI-CS aiming for rapid detection and treatment of cardiogenic shock (19, 31). Patients with AMI are taken to the cath labs when in shock and rapid revascularisation and initiation of MCS is used in patients requiring higher amounts of vasopressors and inotropes in conjunction with increased levels of serum lactate as a sign of systemic hypoperfusion when LV-EF is impaired (32). Impella implantation is initiated during the initial cath lab procedure.

### Patient Population

Based on a previous analysis (19), we calculated a required sample size of *n* = 196 patients to give us 80% power to detect an absolute 10% reduction in mortality with an error of < 0.05. All 202 AMI-CS patients included in the analysis had been supported with an Impella CP at four different shock centres in Germany (University hospitals in Hannover, Bonn and Düsseldorf) and Italy (Padua).

The primary outcome measure of this study was to evaluate if more complete revascularisation defined by a rS of 8 or less would be associated with lower 30 day mortality than less complete revascularisation in AMI-CS patients. In a second step, the same principle was applied to patients with or without Impella implantation prior to PCI.

Since we previously demonstrated higher mortality in AMI-CS patients who suffered cardiac arrest (17, 19) or when hemodynamic support was initiated post-PCI (13, 15, 19), the analysis of the overall cohort was also stratified based on the presence or absence of cardiac arrest and timing of Impella implantation.

### Data Collection and Definitions

Basic demographic data, coronary anatomy, laboratory data and documented complications during in-hospital stay were collected. AMI-CS was defined as hypotension (systolic blood pressure <90 mmHg or need for inotropes or vasopressors to maintain systolic blood pressure >90 mmHg) and evidence of end organ hypoperfusion as indicated by altered mental status, clammy skin, or elevated lactate (>2 mmol/l) after adequate fluid resuscitation. Individual variables were fully available for all patients (32). Bleeding was defined by GUSTO criteria (33) and haemolysis during Impella support was defined as LDH ≥1,000 and haptoglobin <0.3 g/l in 2 consecutive blood samples within 24 h.

### Clinical Follow-Up

Patient follow-up was for the period of hospitalisation, and vital status was determined from medical records. The follow-up of those patients who were discharged from hospital before 30 days was obtained by documents of primary care physicians or rehabilitation hospitals. In case of discharge from hospital or rehabilitation within 30 days, further follow-up was performed by phone. Vital status for 30 days was confirmed in 201/202 patients with the remaining patient discharged home alive on day 11.

### Statistical Analysis

Numbers are given as *n* (%), mean ± standard deviation (SD) for normally distributed variables, or median and interquartile range (IQR) for non-normally distributed variables. Statistical analysis was performed with ANOVA and corrected for multiple comparisons with a Bonferroni correction; Kruskal-Wallis-Test was used for non-parametric tests (17). Chi-square tests were used to compare patient characteristics. Cumulative mortality was estimated by Kaplan-Meier analysis and compared between groups by the log-rank test.

Univariate Cox proportional hazard regression analyses included variables potentially associated with mortality rates were performed to identify factors associated with risk of 30-day mortality. Factors considered included: infarct related artery other than LAD, initial Syntax score, NSTEMI, number of vessels, shock duration until Impella implantation. Then stepwise multivariate Cox regression analyses including variables significantly linked to mortality in the respective univariate analyses (*p* < 0.05) were performed. Analysis for correlation and multicollinearities were performed before multivariate regressions analysis. Results from regression analyses are expressed as hazard ratios (HR) including 95% confidence interval (CI).

Data were analysed using GraphPad Prism 6.0 (GraphPad Software, Inc., La Jolla, CA) and SPSS Statistics 24 (IBM SPSS Statistics 24). A *p* < 0.05 was considered statistically significant.

### Propensity Score Matching

A propensity score matching was performed to minimise confounder bias when comparing 30-day mortality in patients with rS ≤ 8 to patients with rS > 8. Variables related to incomplete revascularization in univariate regressions analysis were taken in to account in propensity score-matching: Infarct related artery other than LAD, initial Syntax score, NSTEMI, numbers of vessels, and shock duration prior to Impella implantation. Matching was realised by a stepwise match on the logit of the estimated propensity score (1:1) between cases and control groups using a nearest neighbour model. Callipers width was equal to 0.2. A balanced distribution of these parameters was achieved. Propensity score-matching was analysed using R program 3.3.3, and SPSS 25 (IBM SPSS Statistics 25).

## Results

### Patient Characteristics

The overall patient population consisted of 202 AMI-CS patients that had been treated with an Impella CP device. Patient characteristics are summarised in Table 1. Mean age in our cohort was 66 ± 11 years and 83% were male. Cardiac arrest had occurred in 94 patients (47%) prior to Impella implantation. Impella was implanted pre-PCI in 96 patients (48%). The type of AMI was STEMI in 60% and NSTEMI in 40%. In general, an average lactate of 5.7 mmol/l and LV-EF of 26% indicate that the patients supported with Impella CP in this analysis were in profound AMI-CS (34, 35).

**Table 1:** Baseline and procedural characteristics of the present prospective cohort.

	**All patients**	**rS-score ≤ 8**	**rS-score > 8**	***p*-value**
	***n* = 202**	***n* =130**	***n* = 72**	**rS≤8 vs. >8**
Age, mean (SD), years	66 ± 11	65 ± 12	67 ± 11	0.1449
Gender- male, *n* (%)	168 (83)	111 (85)	57 (79)	0.2601
Height, mean (SD), cm	174 ± 10	175 ± 11	172 ± 8	0.1321
Weight, mean (SD), kg	84 ± 15	85 ± 16	81 ± 11	0.1099
BMI, mean (SD), kg/m^2^	28.8 ± 13.7	29.3 ± 16.4	27.7 ± 3.7	0.5109
Admission lactate, mean (SD), mmol/L	5.7 ± 4.5	6.0 ± 4.6	5.3 ± 4.3	0.2645
**Pre-existing conditions**				
Hypertension, *n* (%)	133 (66)	86 (66)	47 (65)	0.9005
Diabetes mellitus, *n* (%)	64 (32)	36 (28)	28 (39)	0.1024
Hyperlipidaemia, *n* (%)	68 (34)	40 (31)	28 (39)	0.2443
Smoking, *n* (%)	63 (31)	45 (35)	18 (25)	0.1593
CKD, *n* (%)	43 (21)	23 (18)	20 (28)	0.0842
LV-EF, mean (SD), %	26 ± 11	26 ± 11	27 ± 11	0.6170
Cardiac arrest prior to Impella, *n* (%)	94 (46)	67 (52)	27 (38)	0.0558
ROSC, mean (SD), min	25 ± 20	26 ± 20	24 ± 20	0.7649
Impella pre-PCI, *n* (%)	96 (48)	60 (46)	36 (50)	0.6022
Combination with ECMO, *n* (%)	27 (13)	20 (15)	7 (10)	0.2595
Duration of shock prior to Impella, mean (SD), h	3.3 ± 6.8	2.2 ± 3.3	5.4 ± 10.3	**0.0014**
Infarct location, *n* (%)				**0.0201**
left main	38 (19)	22 (17)	16 (22)	
LAD	106 (52)	77 (59)	29 (40)	
LCX	24 (12)	13 (10)	11 (15)	
RCA	25 (12)	14 (11)	11 (15)	
Bypass graft	9 (4)	4 (3)	5 (7)	
Initial Syntax Score, mean (SD)	29 ± 13	24 ± 12	37 ± 12	** <0.0001**
Residual Syntax Score, mean (SD)	8 ± 10	2 ± 2	19 ± 11	** <0.0001**
TIMI flow at the end of procedure, *n* (%)				** <0.001**
TIMI 0/I	15 (7)	2 (2)	13 (18)	
TIMI II	16 (8)	11 (8)	5 (7)	
TIMI III	171 (85)	117 (90)	54 (75)	
Type of myocardial infarction, *n* (%)				**0.033**
STEMI	121 (60)	85 (65)	36 (50)	
NSTEMI	81 (40)	45 (35)	36 (50)	
Extent of CAD, *n* (%)				**0.001**
1-vessel disease	34 (17)	30 (23)	4 (6)	
2-vessel disease	39 (19)	29 (22)	10 (14)	
3-vessel disease	129 (64)	71 (55)	58 (80)	

*BMI, body mass index; CABG, coronary artery bypass graft; CAD, coronary artery disease; CKD, chronic kidney disease; LAD, left anterior descending coronary artery; LCX, left circumflex coronary artery; LV-EF, left-ventricular ejection fraction; NSTEMI, Non-ST-segement elevation myocardial infarction; PCI, percutaneous coronary intervention; RCA, right coronary artery; ROSC, Return of spontaneous circulation; STEMI, ST-segement elevation myocardial infarction; TIMI, thrombolysis in myocardial infarction.*

*Bold values indicate significant p-values < 0.05*

The most frequent adverse events were the need for renal replacement therapy, bleeding, sepsis and haemolysis (Table 2).

**Table 2:** Thirty-day adverse events.

	**All *n* = 202**
Definite stent thrombosis	2 (1%)
Ischemic stroke	6 (3%)
Haemorrhagic stroke	6 (3%)
Peripheral ischaemia of the leg requiring surgery or intervention	18 (9%)
Haemolysis	67 (33%)
**Bleeding [based on GUSTO definitions** **(****33****)]**	
Lif1e-threatening/severe	20 (10%)
Moderate	45 (22%)
Mild	12 (6%)
Sepsis	73 (36%)
Renal replacement therapy	88 (44%)
Combination with vaECMO	28 (14%)

*vaECMO, veno-arterial extracorporeal membrane oxygenation*.

### Impact of Complete Revascularisation on Mortality

**Figure 1:**
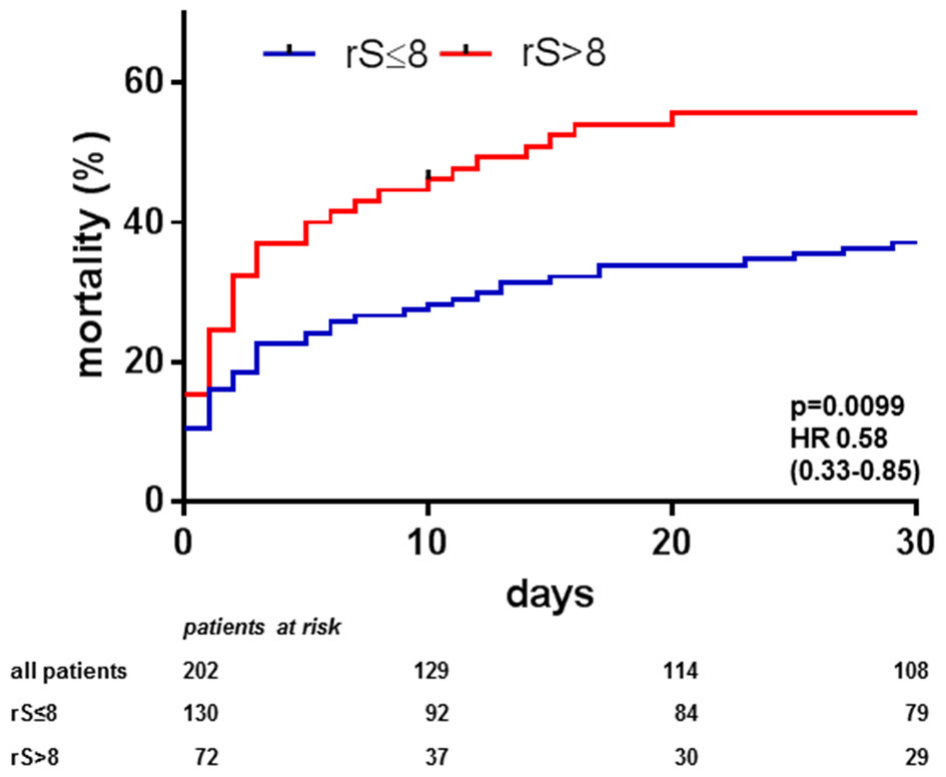
Central illustration. Thirty-day mortality in acute myocardial infarction cardiogenic shock (AMI-CS) on Impella depending on completeness of revascularisation: Observed 30-days mortality in AMI-CS treated with Impella was lower if complete revascularisation defined by an residual Syntax score ≤ 8 was achieved by percutaneous coronary intervention (PCI) compared to less complete revascularisation (rS > 8).

Mean rS was 7.6 in the overall cohort, 130 patients (64%) had an rS ≤ 8, 72 patients (36%) had an rS > 8 with a mean of 18.5 ± 10.6. Patients with rS ≤ 8 had significantly lower 30 day mortality than patients with incomplete revascularisation [rS ≤ 8 37% vs. rS > 8 56%, *p* = 0.0099, HR 0.58, 95%CI (0.33–0.85), Figure 1]. Comparing characteristics of complete compared to incomplete revascularized patients showed that patients with rS ≤ 8 trended to have less pre-existing chronic kidney disease but had a higher rate of pre-Impella cardiac arrest; otherwise, these patients had similar baseline characteristics. In multivariate analysis, only initial Syntax score and duration of shock prior to Impella support remained as independent predictors for incomplete revascularisation (Table 3). As uneven distribution of factors such as type of infarction, number of vessels affected, presence of LAD as culprit, shock duration prior to Impella placement, and baseline Syntax score might have contributed to the observed difference between complete and incomplete revascularisation, we also performed a 1:1 propensity score matching regarding these factors, which reduced the number of cases in the complete revascularisation group from 130 to 56 and from 72 to 56 in the incomplete revascularisation group. The mortality rates were slightly affected (rS ≤ 8: 42% after matching compared to 37% prior to matching; rS > 8: 51% after matching compared to 56% prior to matching), but the strong reduction in cases in the rS ≤ 8 group resulted in a *p* > 0.05. Nevertheless, the trend was in the direction reported prior to propensity score matching (Supplementary Figure 1).

**Table 3:** Uni- and multi-variate analysis of predictors for incomplete revascularisation.

**Parameter**	**Univariate regressions analysis**		**Multivariate regressions analysis**	
	**HR (95% CI)**	***P***	**HR (95%CI)**	***p***
Infarct related artery other than LAD	2.15 (1.19–3.87)	0.01	1.15 (0.56–2.38)	0.698
Initial syntax score	1.09 (1.06–1.13)	<0.001	1.09 (1.06–1.12)	<0.001
NSTEMI	2.01 (1.10–3.67)	0.023	1.41 (0.67–2.93)	0.365
Number of vessels	2.44 (1.51–3.94)	<0.001	1.56 (0.88–2.78)	0.130
Duration shock until Impella implantation	1.08 (1.02–1.15)	<0.001	1.09 (1.01–1.17)	0.024

*LAD, left anterior descending coronary artery; NSTEMI, Non-ST-segement elevation myocardial infarction*.

**Figure 2:**
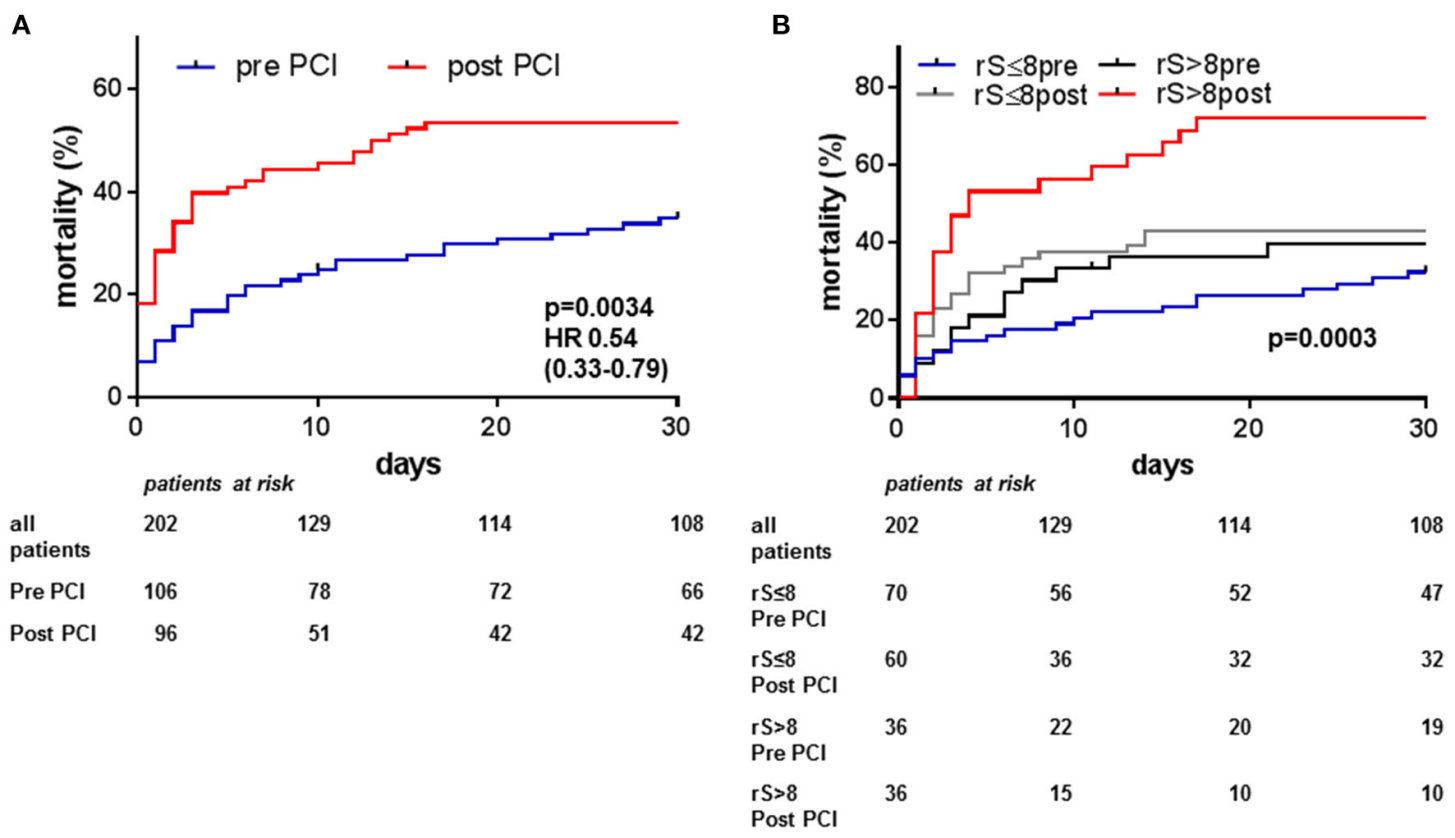
Thirty-day mortality in acute myocardial infarction cardiogenic shock (AMI-CS) on Impella depending on timing of Impella support and completeness of revascularisation: Observed 30-days mortality in AMI-CS treated with Impella was lower if Impella was implanted pre PCI compared to post PCI **(A)**. Lowest mortality was observed in patients receiving Impella pre PCI and achieving complete revascularisation defined by an residual Syntax score (rS) ≤ 8 **(B)**.

Overall 30-day mortality was lower when Impella was implanted pre-PCI (38%, *n* = 40/106) compared to when Impella was implanted post-PCI [57%, *n* = 55/96; *p* = 0.0034, HR 0.54, 95%CI (0.33–0.79), Figure 2A]. While patients receiving Impella post-PCI were younger (64 ± 12 vs. 68 ± 11 years, *p* = 0.0241), they had higher admission lactate levels (6.8 ± 5.0 vs. 4.7 ± 3.7 mmol/l, *p* = 0.0008), longer shock duration prior to Impella (4.2 ± 8.6 vs. 2.5 ± 4.4 h, *p* = 0.0869) and had had cardiac arrest more often prior to implantation (55 vs. 39%, *p* = 0.0186). However, both groups depicted similar LV (pre-PCI 25 ± 11% vs. post-PCI 27 ± 11%, *p* = 0.3420) and renal function (eGFR: pre-PCI 58 ± 28 vs. post-PCI 58 ± 26 ml/min, *p* = 0.8989) prior to support, and success in revascularisation (rS: pre-PCI 8 ± 12 vs. post-PCI 7 ± 9, *p* = 0.2961). Initial Syntax-score was higher in the pre-PCI compared to the post-PCI group (31 ± 14 vs. 26 ± 12, *p* = 0.0033). As circulatory support initiated prior to PCI will improve peri-procedural haemodynamics, we also assessed mortality depending on both co-variates. Patients with both pre-PCI Impella implantation and complete revascularisation had significantly lower mortality than those with incomplete revascularisation and implantation post PCI [33 vs. 72%, HR 0.30, 95%CI (0.10–0.41), *p* < 0.001, Figure 2B].

Duration of shock prior to Impella implantation was shorter in patients with complete revascularisation (rS ≤ 8 2.2 ± 3.3 h vs. rS > 8 5.4 ± 10.3 h, *p* = 0.0014), and most of the benefit of complete revascularisation on mortality was observed in patients with shorter shock duration prior to Impella implantation (Supplementary Figure 2A). Furthermore, mortality could potentially be affected if additive treatment with V-A ECMO were required either due to biventricular failure or need for more potent circulatory support owing to more severe shock. While numerically more patients received ECMELLA support in the completely revascularised group (rS ≤ 8 15% vs. rS > 8 10%, *p* = 0.2595), the overall impact of incomplete revascularisation on mortality was not changed by ECMELLA compared to Impella-only support. In patients with Impella-only support, mortality was significantly lower in the group with rS ≤ 8 compared to less complete revascularisation, and within the ECMELLA group a similar trend was observed (Supplementary Figure 2B). Patients with both pre-PCI Impella implantation and complete revascularisation had significantly lower mortality (33%) than those with incomplete revascularisation and implantation post PCI (72%, *p* < 0.001).

Of the 94 patients who suffered cardiac arrest prior to Impella implantation, 53 died and most common cause of death (*n* = 39, 74%) was due to early haemodynamic instability despite rapid circulatory support. That proportion was larger than in patients without prior cardiac arrest, in whom 39% died due to early haemodynamic failure indicating that late unmasking of underlying anoxic brain damage was not the major driver of increased mortality in patients with cardiac arrest prior to Impella implantation.

## Discussion

In our analysis of 202 AMI-CS patients on Impella support treated by stratified protocols in four high-volume European shock centres, achieving complete revascularisation characterised by a residual Syntax score of 8 or less (21) was associated with lower 30 day mortality than less complete revascularisation. The most promising outcome was observed in patients with [pre PCI] implantation of Impella and complete revascularisation compared to patients with Impella implantation post PCI and incomplete revascularisation.

Recently, the American National Shock Initiative Investigators reported that in their experience multi-vessel PCI in AMI-CS was safe, when patients had been supported with an Impella microaxial flow-pump to rapidly stabilise haemodynamics (23). On first sight, theirs and our results appear to be in contradiction to the randomised CULPRIT-SHOCK trial, in which mortality was even higher when multivessel compared to culprit lesion only PCI was attempted (22). While excess mortality in the multivessel group was mainly driven by anoxic brain damage related to resuscitation prior to revascularisation, the rate of refractory cardiogenic shock was reduced by 12% in the mutlivessel group (22). However, in that trial only 12% of patients received circulatory support by Impella (overall mechanical support by Impella, ECMO, and/or TandemHeart was provided in ~18–19%) and even less were supported by ECMO. The extent of systemic hypoperfusion was similar with an average lactate about 5.0 mmol/l (66% > 2.0 mmol/l) compared to the 5.7 mmol/l (73% > 2.0 mmol/l) in our analysis (22). Recently, that trial and others in stable AMI patients have led to a change in recommendations for revascularisation strategies, whereby guidelines do now prefer culprit-lesion only PCI in AMI-CS (3), but complete revascularisation in stable AMI, contrary to the recommendations given several years before (30). Nevertheless, a large national Korean AMI registry demonstrated lower mortality in multivessel AMI-CS patients when complete compared to culprit-lesion only revascularisation was performed (36). Very recently, a sub-analysis from the CULPRIT-SHOCK trial reported that complete revascularisation was only achieved in roughly 25% of their AMI-CS patients treated using an multi-vessel PCI approach. In their analysis, rS was independently associated with early and late mortality (29). Similarly, findings from the Italian IMP-IT registry using Impella suggested a survival benefit when complete revascularisation was achieved in AMI-CS patients (37).

In patients with stable moderate- and high-risk ACS, incomplete revascularisation with rS above 8 is associated with poor short- and long-term outcome (21). When this parameter was applied to almost 90,000 patients in a meta-analysis, the mortality benefit associated with complete revascularisation was consistent across studies irrespective of revascularization modalities (38). Recently an increasing trend of Impella use over time has been observed along with increased mortality, acute kidney injury, stroke and costs associated with Impella use. Moreover, compared with IABP, Impella was associated with higher mortality, bleeding, acute kidney injury, and stroke. Interestingly, a wide variation in Impella utilisation across hospitals was observed, and hospitals with higher utilisation did not necessarily have better outcomes than lower-use hospitals (39). When trying to perform propensity score matching to patients enrolled in the IABP-Shock II-trial, no benefit had been detected by Impella in AMI-CS, however, the analysis included heterogeneous treatment strategies (40). Recently, retrospective data did suggest that using defined treatment strategies for Impella in AMI-CS could potentially have a beneficial impact on mortality (19, 41). Retrospective observational comparisons between registries and clinical trials inherit the risk of severe selection bias regarding patients selected in clinical practise compared to patients enrolled in clinical trials. For example, of the 202 AMI-CS patients included in the present analysis, 154 (76%) would have fulfilled the inclusion/exclusion criteria of the IABP-Shock II-trial (6), but only 35 (17%) would have qualified for the DanGer-Shock trial (20). Our reported data represent evidence from real clinical practise in AMI-CS, whenever the treating interventional cardiologist felt the need for MCS based on the clinical patient presentation including higher lactate, impaired LV ejection fraction and raised vasopressor demand. As our registry is retrospective, differences in baseline characteristics can influence the allocation to the different treatment strategies as well as the observed outcome. Therefore, we intended to perform a propensity score-matching, after which there was still a trend toward lower mortality in the rS ≤ 8 group, however, the sample size was significantly reduced by the matching and the resulting *p*-value did not achieve statistical significance afterwards. Nevertheless, it has not been our intention to claim superiority of complete revascularisation on MCS over other approaches, we merely wanted to illustrate that results of complete compared to incomplete revascularisation might be different when patients are haemodynamically stabilised during the revascularisation procedure and we should not draw the conclusion that complete revascularisation always results in worse outcome.

In addition to failing cardiac output as a direct consequence of myocardial compromise in AMI-CS, a major contributor to mortality in AMI-CS trials is anoxic brain damage that has occurred prior to hospital admission and prior to insertion of a hemodynamic support device in patients suffering out-of-hospital cardiac arrest [cardiac arrest before enrolment: 28% in IABP-Shock II (6), 54% in CULPRIT-SHOCK (22), 46% in our analysis] as a consequence of AMI-related arrhythmias (17). As a matter of fact, post-arrest brain injury was the most relevant factor in excess mortality between the groups in CULPRIT-SHOCK with an absolute difference of 8.2% in favour of culprit-only PCI. However, regarding manifestation of refractory cardiogenic shock, there was an absolute 8.4% difference in favour of multivessel PCI in the same trial (22). So even while the primary endpoint including mortality was positive toward the culprit-only group based on a non-shock related factor, the more specific shock-related outcome, e.g., refractory cardiogenic shock, was lower in the multivessel-PCI group indicating that complete revacularisation might indeed positively influence shock outcome. Brain injury also highly impacted on the IMPRESS-in-SEVERE-SHOCK trial, in which 92% of patients were post-arrest and which did not demonstrate improved survival on Impella support in a small population of AMI-CS patients (12). While the authors stated that Impella was not effective in CS, not many patients in that study actually had the potential to survive with good neurological outcome. The IABP-Shock II entry criteria (applied in many of the AMI-CS trials) excluded patients who had undergone resuscitation for more than 30 min or were in a coma with fixed dilatation of pupils. In our analysis, pre-implantation cardiac arrest in AMI-CS was associated with a 78% higher 30-day mortality. While in routine treatment a MCS device will not be withheld from AMI-CS patients just because the patient had cardiac arrest before as long as no reliable prediction can be performed to prognosticate neurological outcome, in clinical trials a protocol ensuring exclusion of any comatose post-arrest patients should be employed to test the hypothesis whether mortality can be reduced by standardised use of MCS devices. However, this will exclude a large number of patients and the trial recruitment will be much slower. Nevertheless, such a clear stratified protocol for non-comatose AMI-CS patients testing Impella support compared to standard treatment is currently enrolling, the DanGer-Shock trial (20). Notably, comatose patients after out-of-hospital cardiac arrest and those with prolonged shock duration above 24 h are excluded, in an effort to remove patients who may not derive any benefit from the device due to already established extensive systemic or neurologic damage.

While evidence for MCS use from prospective trials is eagerly awaited and use of IABP in AMI-CS is strongly discouraged, individual decision making is required (32). In order to obtain at least some clarity, we combined our experience from four shock centres with regular Impella use for AMI-CS treatment. When using Impella microaxial flow-pumps to stabilise haemodynamics, we observed better outcome in patients with complete revascularisation compared to those with incomplete revascularisation. Whether complete revascularisation on MCS is superior to current standard treatment, which focuses on culprit lesion only revascularisation without routine circulatory support, needs to be addressed in a randomised-controlled clinical trial.

## Limitations

First, our analysis is based on observational data; so neither controls nor randomised treatment were available. Impella support was initiated whenever the interventional cardiologist felt the need for rapid mechanical circulatory support being justified by elevated lactate levels, impaired LV-ejection fraction on transthoracic echo, and increased vasopressor demand and/or compromised haemodynamics. Obviously, the analysis can only cover patients who survived until implantation of MCS. The results of our analysis are meant to be hypothesis generating given the longer shock duration prior to Impella use and the more complex disease including more NSTEMI in the incomplete revascularisation group. Until prospective trials are conducted, retrospective analyses like ours might, however, suggest using Impella in properly selected patients and appeared at least to be safe if not advantageous to aim for complete revascularisation if the patient is haemodynamically stabilised. While triggering factors for Impella placement might therefore have been different for patients with Impella implanted prior compared to after PCI and those achieving complete compared to incomplete revascularisation, the timing of placement did not significantly affect completeness of revascularisation. Nevertheless, we cannot exclude that higher baseline Syntax score and more patients with TIMI 0/1 flow might have impacted on the overall message, which, however, is in line with a recent sub-analysis from the CULPRIT-SHOCK trial regarding completeness of revascularisation (29). An approach to use propensity score matching for potentially differing baseline parameters indicated a similar trend, but the statistical power was too low owing to the reduction in sample size.

## Conclusions

As long as we are waiting for data from randomised trials, deciding about certain forms of MCS is an individual decision based on the interventionist's experience. While routine use of circulatory support is not suggested, under certain conditions, complete revascularisation supported by an Impella microaxial pump implanted before PCI in AMI-CS might contribute to improved outcomes.

## Data Availability Statement

The raw data supporting the conclusions of this article will be made available by the authors, without undue reservation.

## Ethics Statement

The studies approved by the HAnnover Cardiac Unloading REgistry (HACURE) has a prospective and observational design. The current analysis is in accordance with the Declaration of Helsinki, approved by the ethics committee at Hannover Medical School (#3566-2017). The patients/participants provided their written informed consent to participate in this study.

## Author Contributions

AS, NW, RW, and GT designed the study and drafted the manuscript. J-TS, AZ, JW, CS, and GM critically revised the manuscript. All authors acquired and analysed the data and agree to be accountable for all aspects of the work ensuring that questions related to the accuracy or integrity of any part of the work are appropriately investigated and resolved.

## Conflict of Interest

AS, NW, RW, and GT have received modest lecture fees, honoraria and research grants from Abiomed. The remaining authors declare that the research was conducted in the absence of any commercial or financial relationships that could be construed as a potential conflict of interest.

## Supplementary Material

Supplementary Figure 1 Thirty-day mortality in acute myocardial infarction cardiogenic shock (AMI-CS) on Impella depending on completeness of revascularisation following propensity score matching: Observed 30-days mortality in AMI-CS treated with Impella trended to be lower if complete revascularisation defined by an residual Syntax score ≤ 8 was achieved by percutaneous coronary intervention (PCI) compared to less complete revascularisation (rS > 8) following propensity score matching for type of infarction, number of vessels affected, presence of LAD as culprit, and baseline Syntax score.

Supplementary Figure 2 Thirty-day mortality in acute myocardial infarction cardiogenic shock (AMI-CS) on Impella depending on completeness of revascularisation and extent of cardiogenic shock: Observed 30-days mortality in AMI-CS patients treated with Impella was lower if complete revascularisation defined by an residual Syntax score ≤ 8 was achieved by percutaneous coronary intervention (PCI) compared to less complete revascularisation (rS > 8) independent from the time in shock prior to Impella implantation **(A)** and whether patients were supported by Impella alone or in combination with V-A ECMO (ECMELLA, **B**).

Supplementary Table 1 Characteristics and Outcome depending on time of Impella implantation before vs. after PCI.

## Abbreviations

AMI: acute myocardial infarction
CS: cardiogenic shock
ECMELLA: combination of Impella and ECMO
ECMO: extracorporeal membrane oxygenation
IABP: intraaortic balloon pump
LV: left ventricle/ left ventricular
MCS: mechanical circulatory support
NSTEMI: non-ST-segment elevation myocardial infarction
PCI: percutaneous coronary intervention
STEMI: ST-segment elevation myocardial infarction.
