# Supplementary figures and images for: Complete Revascularisation in Impella-Supported Infarct-Related Cardiogenic Shock Patients Is Associated With Improved Mortality
Source: Front Cardiovasc Med. 2021 Jul 9;8:678748. doi: 10.3389/fcvm.2021.678748 (PMC8299360; doi:10.3389/fcvm.2021.678748)

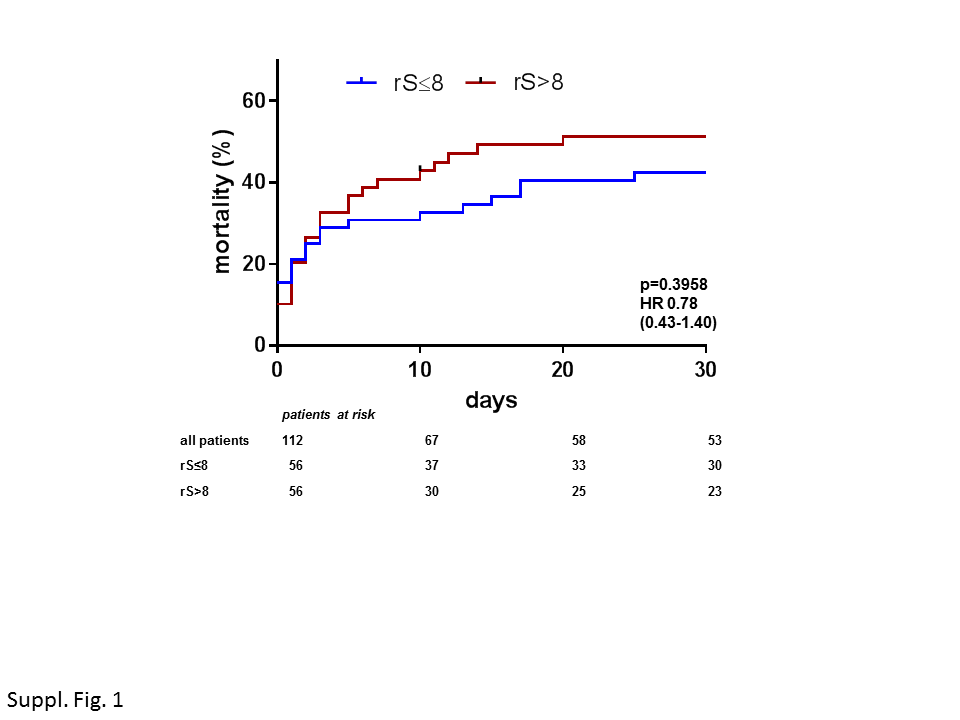

Supplement: Supplementary Figure 1 — Thirty-day mortality in acute myocardial infarction cardiogenic shock (AMI-CS) on Impella depending on completeness of revascularisation following propensity score matching: Observed 30-days mortality in AMI-CS treated with Impella trended to be lower if complete revascularisation defined by an residual Syntax score ≤ 8 was achieved by percutaneous coronary intervention (PCI) compared to less complete revascularisation (rS > 8) following propensity score matching for type of infarction, number of vessels affected, presence of LAD as culprit, and baseline Syntax score. [file Image_1.tif]

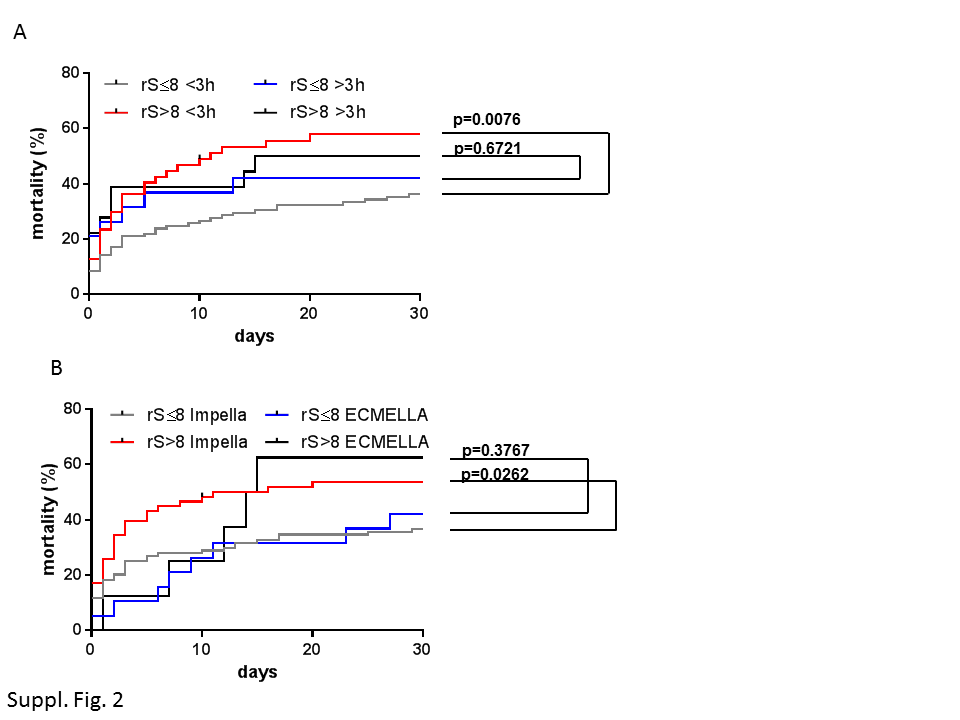

Supplement: Supplementary Figure 2 — Thirty-day mortality in acute myocardial infarction cardiogenic shock (AMI-CS) on Impella depending on completeness of revascularisation and extent of cardiogenic shock: Observed 30-days mortality in AMI-CS patients treated with Impella was lower if complete revascularisation defined by an residual Syntax score ≤ 8 was achieved by percutaneous coronary intervention (PCI) compared to less complete revascularisation (rS > 8) independent from the time in shock prior to Impella implantation (A) and whether patients were supported by Impella alone or in combination with V-A ECMO (ECMELLA, B). [file Image_2.tif]
