# Supplementary material for: Complete Revascularisation in Impella-Supported Infarct-Related Cardiogenic Shock Patients Is Associated With Improved Mortality
Source: Front Cardiovasc Med. 2021 Jul 9;8:678748. doi: 10.3389/fcvm.2021.678748 (PMC8299360; doi:10.3389/fcvm.2021.678748)
Supplement: Supplementary Table 1 — Characteristics and Outcome depending on time of Impella implantation before vs. after PCI. [file Table_1.docx]

**Suppl. Table 1: Characteristics and Outcome depending on time of Impella implantation before vs after PCI**

|  | Impella pre-PCI  n= 106  mean±SD, median [IQR], n (%) | Impella post PCI  n=96  mean±SD, median [IQR], n (%) | p-value  pre vs. post |
| --- | --- | --- | --- |
| Age, mean (SD), years | 68 ± 11 | 64 ± 12 | 0.0241 |
| Cardiac arrest prior to Impella | 41 (39%) | 53 (55%) | 0.0186 |
| Admission lactate, mean (SD), mmol/L | 4.7 ± 3.7 | 6.8 ± 5.0 | 0.0008 |
| eGFR, mean (SD), ml/min | 58 ± 28 | 58 ± 26 | 0.8989 |
| LV-EF prior to Impella, mean (SD), % | 25 ± 11 | 27 ± 11 | 0.3420 |
| Duration of shock prior to Impella, mean (SD), hrs | 2.5±4.4 | 4.2±8.6 | 0.0869 |
| Initial Syntax Score | 31 ±14 | 26 ± 12 | 0.0033 |
| Residual Syntax Score | 8 ±12 | 7 ± 9 | 0.2961 |

eGFR-estimated glomerular filtration rate; LV-EF-left-ventricular ejection fraction; SAPS II-Simplified Acute Physiology Score-II
